# Supplementary figures and images for: Empirical analysis and modeling of Argos Doppler location errors in Romania
Source: PeerJ. 2019 Jan 31;7:e6362. doi: 10.7717/peerj.6362 (PMC6360076; doi:10.7717/peerj.6362)

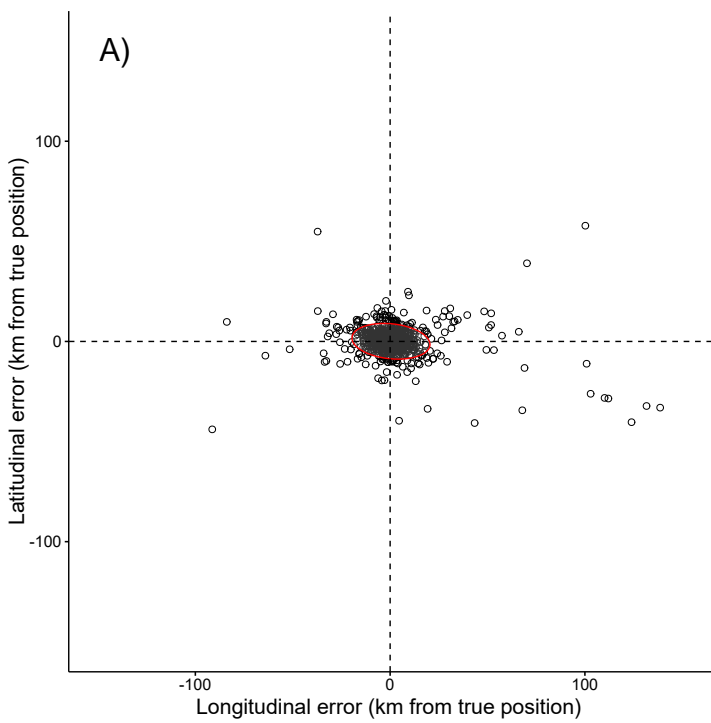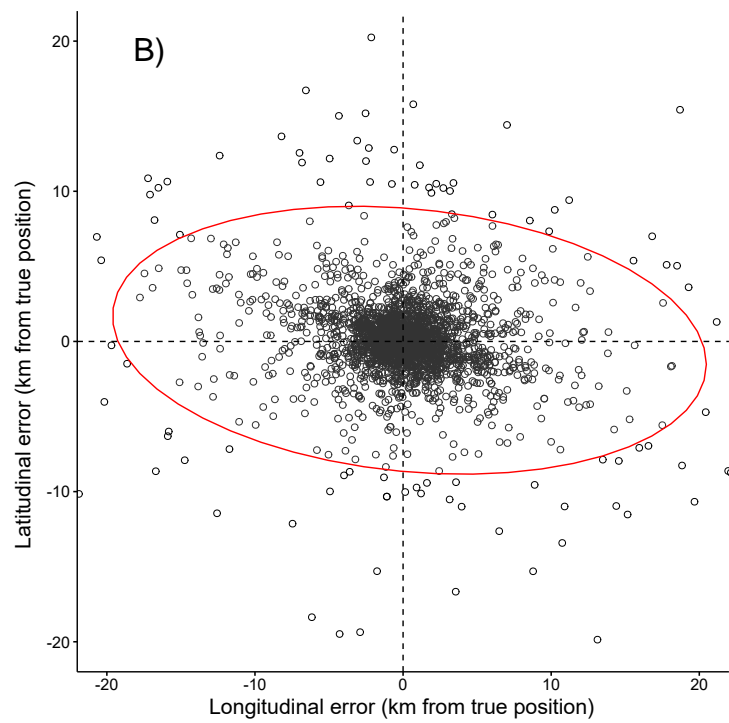

Supplement: Supplemental Information 2 [file peerj-07-6362-s002.pdf]

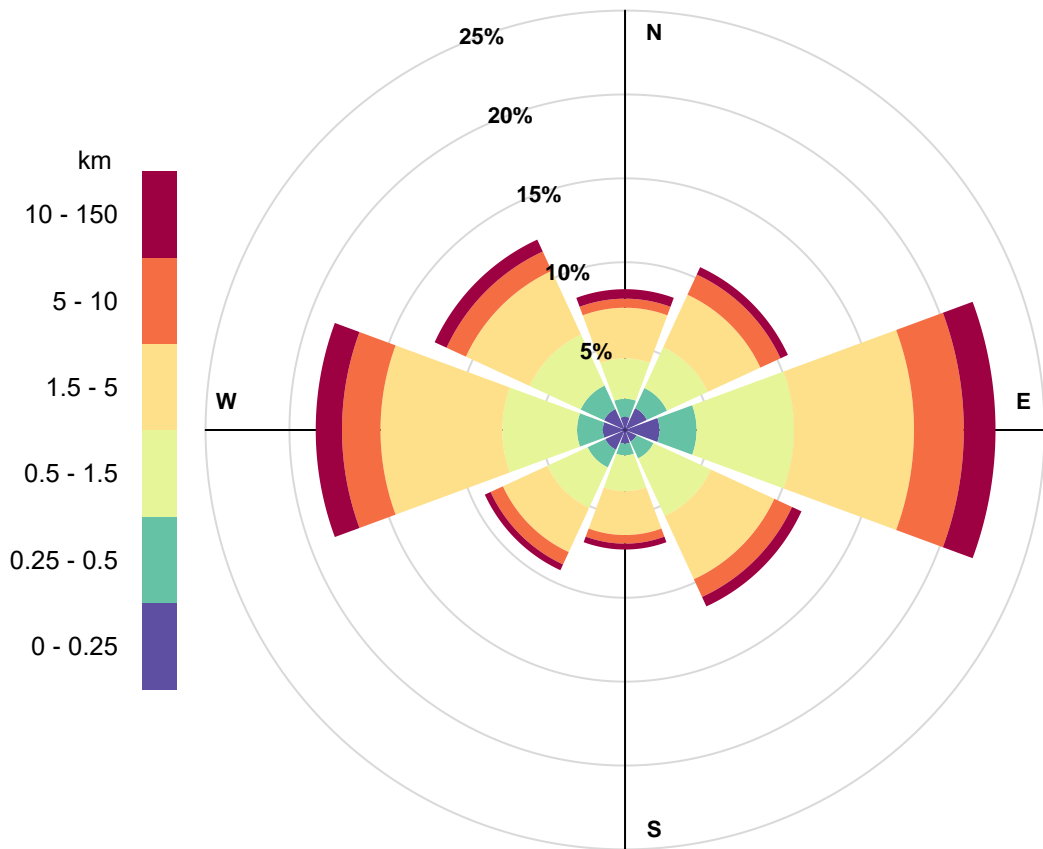

Supplement: Supplemental Information 3 [file peerj-07-6362-s003.pdf]

NoF DAR2 DAR5 DAR10 DAR15

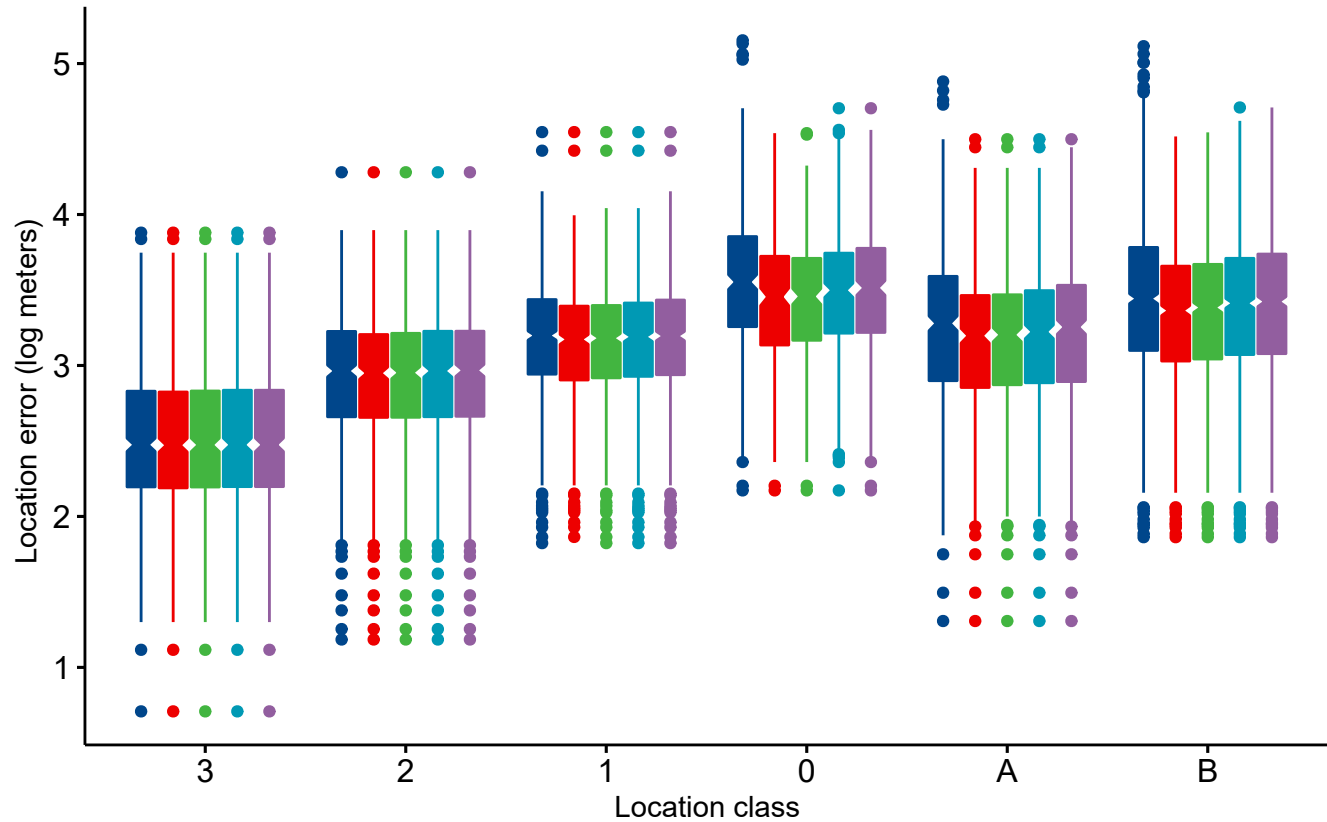

Supplement: Supplemental Information 4 — NoF = unfiltered data, DAR 2 = Douglas Argos DAR with MAXREDUN = 2 km, DAR 5 = Douglas Argos DAR with MAXREDUN = 5 km, DAR 10 = Douglas Argos DAR with MAXREDUN = 10 km, DAR 15 = Douglas Argos DAR with MAXREDUN = 15 km. [file peerj-07-6362-s004.pdf]
