# Supplementary material for: Empirical analysis and modeling of Argos Doppler location errors in Romania
Source: PeerJ. 2019 Jan 31;7:e6362. doi: 10.7717/peerj.6362 (PMC6360076; doi:10.7717/peerj.6362)
Supplement: Supplemental Information 5 — Place = trial sites, motion = static, low-speed, high-speed, TRI = terrain ruggedness index, 1 = baseline model, AICc = Akaike’s Information Criterion corrected for small sample size. The random effects part includes reception points nested in the satellite providing the respective location. [file peerj-07-6362-s005.docx]

| Model fixed factors | df | AIC_c_ | AIC_c_wt | weight |
| --- | --- | --- | --- | --- |
| Place + Motion | 9 | 4831.7 | 0.00 | 0.685 |
| Motion | 6 | 4834.1 | 2.44 | 0.202 |
| Place + Motion + TRI | 10 | 4835.3 | 3.61 | 0.113 |
| Place × Motion | 15 | 4846.9 | 15.19 | < 0.001 |
| Place × Motion + TRI | 16 | 4850.5 | 18.83 | < 0.001 |
| 1 (null model) | 4 | 5078.7 | 247.07 | < 0.001 |
| Place | 7 | 5085.2 | 253.53 | < 0.001 |
