# Supplementary material for: Empirical analysis and modeling of Argos Doppler location errors in Romania
Source: PeerJ. 2019 Jan 31;7:e6362. doi: 10.7717/peerj.6362 (PMC6360076; doi:10.7717/peerj.6362)
Supplement: Supplemental Information 6 [file peerj-07-6362-s006.docx]

| Location class | % locations retained | Mean error (stdev), meters | 68^th^ percentile of errors, meters | Mean error longitude (stdev), meters | Mean error latitude, (stdev) meters | % locations in error ellipse retained | % locations out of error ellipse retained |
| --- | --- | --- | --- | --- | --- | --- | --- |
| LC 3 | 96.02 | 567.53 (798.32) | 507.96 | 456.28 (744.12) | 249.71 (366.92) | 96.36 | 95.98 |
| LC 2 | 90.38 | 1198.12 (1295.45) | 1337.51 | 936.48 (1100.64) | 570.29 (835.83) | 95.83 | 90.12 |
| LC 1 | 85.46 | 1996.58 (2351.56) | 2108.89 | 1589.89 (2039.98) | 915.01 (1408.85) | 97.37 | 84.75 |
| LC 0 | 68.35 | 3907.59 (4124.47) | 4667.51 | 3233.44 (4018.79) | 1646.00 (1719.10) | 97.37 | 65.86 |
| LC A | 80.00 | 2441.70 (3190.12) | 2534.84 | 1844.42 (2948.79) | 1183.93 (1622.59) | 84.00 | 79.56 |
| LC B | 82.66 | 3554.50 (3880.18) | 3641.73 | 2649.74 (3465.52) | 1862.83 (2275.08) | 89.32 | 84.29 |
| Total | 84.35 | 2313.51  (3134.67) | 2252.89 | 1784.746 (2788.20) | 1126.42  (1715.18) | 90.93 | 82.26 |
