# Supplementary material for: Empirical analysis and modeling of Argos Doppler location errors in Romania
Source: PeerJ. 2019 Jan 31;7:e6362. doi: 10.7717/peerj.6362 (PMC6360076; doi:10.7717/peerj.6362)
Supplement: Supplemental Information 7 [file peerj-07-6362-s007.docx]

| Location class | % locations retained | Mean error (stdev), meters | 68^th^ percentile of errors, meters | Mean error longitude (stdev), meters | Mean error latitude, (stdev) meters | % locations in error ellipse retained | % locations out of error ellipse retained |
| --- | --- | --- | --- | --- | --- | --- | --- |
| LC 3 | 97.35 | 580.40 (806.93) | 525.80 | 466.73 (747.80) | 257.20 (379.84) | 98.18 | 97.25 |
| LC 2 | 97.88 | 1239.32  (1289.91) | 1392.26 | 976.06 (1106.08) | 583.57 (825.63) | 95.83 | 97.98 |
| LC 1 | 96.74 | 2201.28 (2478.88) | 2268.96 | 1765.88 (2163.2) | 998.19 (1480.09) | 100 | 96.54 |
| LC 0 | 90.42 | 4783.05 (5281.87) | 5255.86 | 3949.20 (5050.06) | 2020.31 (2360.83) | 95.24 | 89.82 |
| LC A | 93.86 | 2763.46 (3247.40) | 2824.62 | 2063.67 (2993.83) | 1365.62 (1757.32) | 88 | 94.51 |
| LC B | 92.92 | 4095.57 (4665.61) | 4331.42 | 3061.02 (4260.98) | 2130.64 (2541.18) | 94.82 | 92.18 |
| Total | 94.82 | 2702.64  (3732.31) | 2629 | 2090.37 (3358.29) | 1308.30 (1965.46) | 94.98 | 94.79 |
